# Immune Axonal Neuropathies Associated With Systemic Autoimmune Rheumatic Diseases

**DOI:** 10.3389/fphar.2021.610585

**Published:** 2021-04-14

**Authors:** Delia Tulbă, Bogdan Ovidiu Popescu, Emilia Manole, Cristian Băicuș

**Affiliations:** ^1^ Department of Neurology, Colentina Clinical Hospital, Bucharest, Romania; ^2^ Colentina-Research and Development Center, Colentina Clinical Hospital, Bucharest, Romania; ^3^ “Carol Davila” University of Medicine and Pharmacy, Bucharest, Romania; ^4^ Laboratory of Cell Biology, Neurosciences and Experimental Myology, “Victor Babeș” National Institute of Pathology, Bucharest, Romania; ^5^ Department of Internal Medicine, Colentina Clinical Hospital, Bucharest, Romania

**Keywords:** immune axonal neuropathy, vasculitic neuropathy, sensorimotor polyneuropathy, mononeuritis multiplex, connective tissue disease, systemic vasculitis, systemic autoimmune rheumatic disease, small fiber neuropathy

## Abstract

Immune axonal neuropathies are a particular group of immune-mediated neuropathies that occasionally accompany systemic autoimmune rheumatic diseases such as connective tissue dissorders and primary systemic vasculitides. Apart from vasculitis of vasa nervorum, various other mechanisms are involved in their pathogenesis, with possible therapeutic implications. Immune axonal neuropathies have highly heterogeneous clinical presentation and course, ranging from mild chronic distal sensorimotor polyneuropathy to severe subacute mononeuritis multiplex with rapid progression and constitutional symptoms such as fever, malaise, weight loss and night sweats, underpinning a vasculitic process. Sensory neuronopathy (ganglionopathy), small fiber neuropathy (sensory and/or autonomic), axonal variants of Guillain-Barré syndrome and cranial neuropathies have also been reported. In contrast to demyelinating neuropathies, immune axonal neuropathies show absent or reduced nerve amplitudes with normal latencies and conduction velocities on nerve conduction studies. Diagnosis and initiation of treatment are often delayed, leading to accumulating disability. Considering the lack of validated diagnostic criteria and evidence-based treatment protocols for immune axonal neuropathies, this review offers a comprehensive perspective on etiopathogenesis, clinical and paraclinical findings as well as therapy guidance for assisting the clinician in approaching these patients. High quality clinical research is required in order to provide indications and follow up rules for treatment in immune axonal neuropathies related to systemic autoimmune rheumatic diseases.

## Introduction

Autoimmune diseases are a broad group of conditions characterized by chronic activation of the immune system that eventually leads to tissue inflammation and damage (Doria et al., 2012). In contrast to autoinflammatory disorders entirely mediated by the innate immune system, autoimmune diseases imply dysregulation of both innate and adaptive immunity, yet the injury is mediated by adaptive immune responses (Doria et al., 2012; Firestein et al., n.d.). However, a less restrictive demarcation might be appropriate since conditions such as Behçet’s disease (BD) share both autoimmune and autoinflammatory features, underpinning the possibility of an autoreactivity spectrum that encompasses autoimmune diseases at one end and autoinflammatory diseases at the other (Firestein et al., n.d.).

Autoimmune diseases are classified into organ-specific and systemic conditions, depending on their expansion (Firestein et al., n.d.). In systemic autoimmune disorders, autoreactivity targets ubiquitous self-antigens, leading to autoantibodies and/or T cells that mediate end-organ injury (Firestein et al., n.d.). Since the musculoskeletal system is often targeted, most of them are considered systemic autoimmune rheumatic diseases (SARDs) and include systemic lupus erythematosus (SLE), rheumatoid arthritis (RA), primary Sjögren’s syndrome (SS), antiphospholipid syndrome (aPL), systemic sclerosis (SSc), sarcoidosis and systemic vasculitides (Doria et al., 2012; Firestein et al., n.d.; Starshinova et al., 2019). According to the type of the vessels involved, systemic vasculitides are further classified into predominantly large vessel vasculitides (e.g., giant cell arteritis), predominantly medium vessel vasculitides (e.g., polyarteritis nodosa (PAN)) and predominantly small vessel vasculitides (e.g., antineutrophil cytoplasmic antibody (ANCA)-associated vasculitides and cryoglobulinemia) (Jennette et al., 2012). Another classification identifies primary systemic vasculitides (e.g., ANCA-associated vasculitides) and secondary systemic vasculitides that occur in the setting of other SARDs (e.g., lupus vasculitis, rheumatoid vasculitis, sarcoid vasculitis), various infections, drugs, malignancies, inflammatory bowel disease and hypocomplementemic urticarial vasculitis syndrome (Collins et al., 2010; Sampaio et al., 2011; Collins, 2012; Jennette et al., 2012).

Systemic autoimmune rheumatic diseases occasionally involve the nervous system. Moreover, the presence of central or peripheral nervous system dysfunction of unknown cause can assist the diagnosis of SARDs, as pointed out by the classification criteria designed for some of these disorders. For instance, the 2012 SLICC Systemic Lupus Erythematosus Criteria refer to “mononeuritis multiplex, peripheral or cranial neuropathy” as a clinical criterion for lupus (Petri et al., 2012); the 1990 ACR Classification Criteria for Polyarteritis Nodosa (Lightfoot Jr. et al., 1990) or Churg-Strauss Syndrome (Masi et al., 1990) mention “mononeuropathy or polyneuropathy” likewise. However, the inaccurate definition of terms–i.e. “peripheral neuropathy” does not specifically refer to immune axonal neuropathy, which is the main peripheral nervous system involvement in SARDs–reveals the diagnostic limits of these criteria (Collins et al., 2013).

Immune axonal neuropathies are a heterogeneous group of immune-mediated peripheral neuropathies that target the axons, showing absent or reduced nerve amplitudes with normal latencies and conduction velocities on nerve conduction studies, in contrast to demyelinating neuropathies (Bril and Katzberg, 2014). They are linked to various conditions such as SARDs, monoclonal gammopathy, celiac disease, inflammatory bowel disease, paraneoplastic syndromes and infections (Bril and Katzberg, 2014). Vasculitic neuropathies are a distinct group of immune axonal neuropathies that appear in the setting of a vasculitis. Inflammation of vasa nervorum leads to fibrinoid necrosis, with multifocal nerve infarction and accumulating disability (Sampaio et al., 2011). Their recognition is important since they do not usually respond to intravenous (IV) immunoglobulin and require a higher level of immunosuppression straightaway.

We aim to review immune axonal neuropathies associated with SARDs, with a special focus on vasculitic neuropathies encountered in systemic vasculitides, both primary and secondary to other SARDs. We have briefly discussed about BD (and BD-associated neuropathy), which is classified either as SARD or autoinflammatory disorder. We have not included secondary systemic vasculitides (other than SARDs), non-systemic/localized vasculitides (i.e. non-systemic vasculitic neuropathy, localized cutaneous/neuropathic vasculitis), demyelinating neuropathies and neuropathies with other mechanisms (e.g. nerve entrapment). Considering the lack of validated diagnostic criteria and evidence-based treatment protocols, a comprehensive review on etiopathogenesis, clinical and paraclinical findings as well as treatment guidance are offered in order to assist the clinician in approaching the patients with immune axonal neuropathies related to SARDs.

## Epidemiology

There is a scarcity of data regarding the incidence and prevalence of immune axonal neuropathies associated with SARDs, but some information can be drawn indirectly from other epidemiological findings. For instance, systemic vasculitides, both primary and secondary, have an annual incidence of 60–140/million (Blaes, 2015) and up to 60–70% of patients with systemic vasculitis develop neuropathy (Gwathmey et al., 2014). Up to 30% of elderly patients with progressive, severe and painful peripheral nervous system involvement might have vasculitic neuropathy (Vrancken and Said, 2013) and 1% of nerve biopsy specimens from patients with cryptogenic neuropathy display vasculitic features (Vrancken and Said, 2013; Blaes, 2015). However, since these findings pertain to the whole group of vasculitides (including non-systemic/localized vasculitides and systemic vasculitides secondary to infections, drugs, malignancies, etc.), it is difficult to obtain accurate and relevant information about vasculitic neuropathies strictly related to SARDs. Moreover, the broad interval of vasculitic neuropathy frequency reported in SARDs patients, ranging from 15 to 70% in RA, 65–80% in eosinophilic granulomatosis with polyangiits (EGPA), 5–50% in granulomatosis with polyangiits (GPA) and 6–75% in microscopic polyangiitis (MPA) reflects the heterogeneity in population and diagnostic means (either clinical or electrophysiological testing) among studies (Snoussi et al., 2019). Epidemiological data about other types of immune axonal neuropathies related to SARDs are even more scarce.

## Etiopathogenesis

The nutrient supply of peripheral nerves is provided by both neuronal cell bodies (which synthesize proteins and transfer them down the axons through the microtubules) and blood vessels, the latter being particularly important in nerves with long axons (Ishibe et al., 2011). An impairment at any of these levels might damage the nerve and cause malfunctioning (e.g., ischemia due to vasa nervorum thrombosis/blood flow restriction usually manifests as mononeuritis multiplex, inflammatory damage of axons induces axonal sensorimotor polyneuropathy, inflammatory injury of dorsal root ganglia neurons leads to sensory neuronopathy (ganglionopathy), whereas immunological/metabolic changes elicit small nerve fiber degeneration with subsequent small fiber neuropathy).

### Vasa Nervorum Vasculitis

**FIGURE 1:**
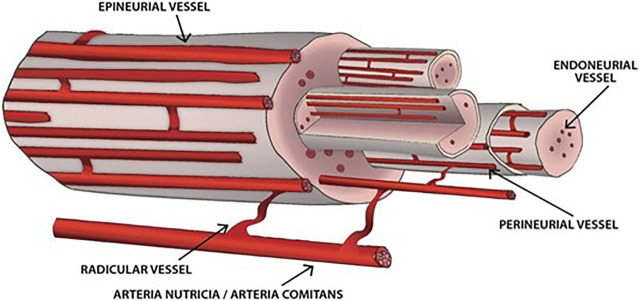
Vasa nervorum. The extrinsic vessels derive from either arteriae nutriciae or arteria comites and branch into radicular vessels. The intrinsic vessels are supplied by radicular vessels, run longitudinally along the nerve and comprise epineurial, perineurial and endoneurial vessels. Various anastomoses between and within each of these structures arise.

Vasa nervorum (the vasculature of peripheral nerves) (Figure 1) are a complex vessel network designed to fulfill the structural and functional metabolic requirements of the nerves and to maintain homeostasis within the endoneurial microenvironment, as part of the blood nerve barrier (which involves the perineurial cellular layers and the endoneurial capillary endothelium) (Mizisin and Weerasuriya, 2011). Vasa nervorum consist of two distinct systems, the extrinsic and intrinsic vessels, with various anastomoses between and within each of them. The extrinsic system (extraneural vasa nervorum) includes regional arteries, either arteriae nutriciae from adjacent large muscular arteries or arteriae comites from musculocutaneous/fascial arteries, that branch into radicular vessels responsible for supplying the intraneural vessels (Boissaud-Cooke et al., 2015). As they insert segmentally into the epineurium, a longitudinal plexus of arterioles takes shape, marking the outer layer of the intrinsic system (intraneural vasa vasorum) (Boissaud-Cooke et al., 2015). While reaching deeper into the epineurium, a further multiplanar branching occurs, generating the terminal arterioles that penetrate the perineurium and cross it mainly obliquely (Boissaud-Cooke et al., 2015). They eventually end up as an intrafascicular endoneurial vascular plexus, running along the longitudinal axis of the nerve, yet with multidirectional branching and anastomoses (Boissaud-Cooke et al., 2015). The endoneurial capillary bed consists of particular capillaries—with large diameter resembling that of postcapillary venules, increased intercapillary distance and tight endothelial junctions with sporadic open interendothelial gaps (Mizisin and Weerasuriya, 2011), outlining the blood nerve interface (Ishibe et al., 2011; Boissaud-Cooke et al., 2015). This intricate structural and functional organization of the vasa nervorum coupled with the low compliance of the perineurium - preventing increases in intrafascicular volume - might explain the vulnerability of peripheral nerves to slight increases in capillary permeability: the endoneurial edema briskly raises the hydrostatic pressure and leads to compression of transperineurial vessels and subsequent reduction in blood flow and ischemia (Mizisin and Weerasuriya, 2011; Boissaud-Cooke et al., 2015).

Systemic vasculitides (either primary or secondary to other SARDs) can affect the vasa nervorum, leading to inflammatory damage of vessel walls and blood flow restriction/thrombosis responsible for nerve ischemia (Sampaio et al., 2011; Collins et al., 2013). Considering the dimensions of the blood vessels in the peripheral nerves (approximately 10–300 μm), it is not surprising that vasculitic neuropathies are mainly confined to medium and small vessel vasculitides (Vrancken and Said, 2013; Naddaf and Dyck, 2015). The inflammation usually occurs in the small arteries and large arterioles of the epineurium and perineurium, as opposed to the non-systemic vasculitic neuropathy that involves the endoneurial microvessels (Collins et al., 2013; Gwathmey et al., 2014). Although the pathogenesis of systemic vasculitides mainly implies a humoral immune response, the vasa nervorum display a cellular immune-mediated reaction with vascular infiltrates of CD4+/helper and CD8+/cytotoxic T cells and antigen-presenting cells (mainly macrophages) in the epineurial vessel walls (Jennette et al., 2012). A pathogenic model centered on T cells with the following sequences has been proposed: autoreactive T cells are recruited to the peripheral nervous system, recognize the antigens presented by macrophages, endothelial cells and Schwann cells, undergo activation by cell adhesion molecules and chemotactic cytokines and ultimately mature into or recruit cytotoxic T cells that mediate the destruction of vessel walls (Sampaio et al., 2011; Collins et al., 2013). An ongoing inflammatory milieu ensues as the macrophages exhibit upregulation of the inducible costimulator ligand that binds to the highly expressed inducible costimulator (a CD28-like molecule engaged in T-cell activation) on effector memory T cells, hence restimulating activated T cells (Collins et al., 2013). Although of lesser importance, humoral immune mechanisms also contribute to vasa nervorum vasculitis, as indicated by the deposits of immunoglobulin and complement in epineurial vessel walls that imply either *in situ* formation of immune complexes or deposition of circulating ones, with consecutive activation of the complement system and recruitment of phagocytes (Collins et al., 2013). The involvement of ANCA in the pathogenesis of vasculitic neuropathy is doubtful: more than half of the ANCA-negative EGPA patients have peripheral neuropathy, whereas ANCA are rarely detected in the most common vasculitic neuropathy, namely nonsystemic vasculitic neuropathy (Collins et al., 2013). Furthermore, the rich immune complex deposits found in vasculitic neuropathies do not match the pauci-immune states of ANCA-associated vasculitides (Collins et al., 2013) and the prevalence of vasculitic neuropathy is similar in seropositive and seronegative ANCA-associated vasculitides (Collins, 2012).

### Immune and Metabolic Abnormalities

Apart from the vasculitis of vasa nervorum that leads to ischemic damage of the axon, various other pathogenic mechanisms have been observed in patients with immune axonal neuropathy related to SARDs, particularly immune/inflammatory abnormalities and metabolic changes (Bril and Katzberg, 2014; Snoussi et al., 2019). In SLE, local inflammatory changes (i.e. increased vascular permeability and cellular trafficking) are mediated by neuropeptides such as substance P, calcitonin gene-related protein, nitric oxide and chemokines (Imam et al., 2019). Inflammatory molecules released from activated immune cells induce peripheral sensitization and hyperalgesia by targeting sensory neurons (Imam et al., 2019). Additionally, a direct autoantibody-induced aggression is supposedly pathogenic (Mizisin and Weerasuriya, 2011). Antiphospholipid antibodies are linked to severe neural lesions (Snoussi et al., 2019) and are thought to induce ischemic damage by thrombosis of vasa nervorum (Imam et al., 2019) or vasculitis (Santos et al., 2010; Fleetwood et al., 2018). Antigenic determinants of the myelin phospholipids might be targets for anticardiolipin antibodies, leading to demyelinating neuropathies, as suggested by Santos et al. (Santos et al., 2010). Moreover, it has been hypothesized that anticardiolipin antibodies elicit an immune response by binding to nodal axonal epitopes, which might be responsible for the axonal variants of Guillain-Barré syndrome (GBS) reported in SLE patients (Santiago-Casas et al., 2013). In SS, small fiber neuropathy is probably the result of immune-mediated disruption of small unmyelinated or thinly myelinated fibers by inflammatory perivascular infiltrates, with elevated local proinflammatory cytokines such as IL-6, IL-8, TNF-α and IL-1β (Hoeijmakers et al., 2012; Bril and Katzberg, 2014), whereas sensory neuronopathy (ganglionopathy) occurs in the setting of direct inflammatory damage of dorsal root ganglia neurons mediated by CD8 T lymphocytes (Martinez et al., 2012). In SSc, the mechanism of neuropathy has not been elucidated, but acral vasospasm secondary to Raynaud phenomenon might be involved in its etiopathogenesis (Paik et al., 2016). Infiltration of the epineurium and perineurium by sarcoid granuloma supposedly causes axonal damage and neuropathy in sarcoidosis (Pirau and Lui, 2020), whereas both humoral and cellular abnormal immune responses might play a role in the development of neuropathy in BD (Emam, 2019). Apart from the vasculitis of vasa nervorum, eosinophilic infiltration and granuloma formation are involved in the pathogenesis of EGPA-related neuropathy (Collins, 2012). In cryoglobulinemia, either cryoglobulin precipitation with subsequent vessel occlusion or immune complex deposition along myelin occurs (Collins, 2012). A direct pathogenic role of antisulfatide antibodies and GM1-ganglioside antibodies has also been proposed (Blaes, 2015). Up to 32% of patients with connective tissue disorders have increased levels of serum anti-nerve growth factor antibodies, which correlate with high disease activity and severe nervous system manifestations (Snoussi et al., 2019). On the other hand, an increased expression of nerve growth factor occurs in painful vasculitic neuropathies (Blaes, 2015).

### Axonal Degeneration

The axons play a significant role in long-distance neuronal communication through action potential initiation and propagation and action potential-mediated transmitter release. The vasculitic neuropathies lead to nerve ischemia and consecutive focal and asymmetrical axonal loss in a specific spatial distribution–it is likely to be centrofascicular in the proximal areas and multifocal/diffuse in distal ones because of twisted nerve fibers (Sampaio et al., 2011; Collins et al., 2013). Axonal degeneration occurs mostly in large sensory and motor myelinated fibers (Sampaio et al., 2011; Suppiah et al., 2011) and is responsible for the length-dependent pattern of the disease, affecting the longest nerves from distal extremities. There is a reciprocal link between the axons and the vasa nervorum. On one hand, as already mentioned, inflammatory damage of vessel walls and blood flow restriction promote axonal ischemia and loss. Since axonal degeneration is associated with increased permeability of the perineurium, it has been suggested that the axon might be responsible for producing diffusible factors that maintain the tight intercellular junctions (by regulating the synthesis of intercellular junctional proteins) in the perineurium (Weerasuriya and Mizisin, 2011). This could indicate that axonal degeneration is not merely a final path in the etiopathogenic process of vasculitic neuropathy. However, this hypothesis needs to be tested.

## Clinical Presentation

In immune axonal neuropathy related to SARDs, the clinical presentation ranges from asymptomatic to very aggressive forms of neuropathy leading to significant disability (Blaes, 2015).

### Clinical Patterns of Immune Axonal Neuropathy in Systemic Autoimmune Rheumatic Diseases

Sensorimotor polyneuropathy is the main phenotype of neuropathy in collagen vascular diseases and the second most common presentation of vasculitic neuropathy in primary systemic vasculitides (Bril and Katzberg, 2014; Blaes, 2015). These patients present with chronic distal symmetrical sensorimotor symptoms. A pure sensory or motor presentation is rarely found (Blaes, 2015).

Mononeuritis multiplex is the classical presentation of vasculitic neuropathy, occurring in up to 65% of cases (Blaes, 2015). It is defined as an “acute or subacute involvement of multiple individual nerves serially or almost simultaneously” (Ropper et al., 2014). It usually presents with painful sensorimotor deficit in the distribution of a single peripheral nerve (Bril and Katzberg, 2014), the peroneal and tibial nerves being mostly affected, followed by the ulnar nerve (Blaes, 2015). The simultaneous or sequential multifocal involvement with overlapping clinical deficits might give the appearance of an asymmetrical polyneuropathy (Collins et al., 2013), but constitutional symptoms (i.e. fever, fatigue, weight loss) should raise suspicion of a vasculitic process. Constitutional symptoms accompany neurological dysfunction in up to 80% of patients with systemic vasculitis (Blaes, 2015).

Pure sensory neuropathy may also be a presentation of sensory neuronopathy (ganglionopathy) in SS or SLE (Martinez et al., 2012; Bril and Katzberg, 2014; Lefter et al., 2020). The manifestations of sensory neuronopathy are usually multifocal and spread toward both proximal and distal regions of the limbs. They comprise all sensory modalities (proprioception, vibration sense, fine touch, pain, temperature), with prominent gait ataxia as well as widespread areflexia and possible pseudoathetosis, pain and allodynia (Martinez et al., 2012). An acute onset with disabling manifestations is often encountered in SS-related ganglionopathy (Martinez et al., 2012). Pure sensory symptoms might also arise as a consequence of *small fiber neuropathy* in SS or sarcoidosis (Levine, 2018), usually with positive manifestations such as paresthesia, dysesthesia, hyperpathia, allodynia and hyperalgesia that are more severe in the evening and have a symmetrical length-dependent distribution–beginning in the feet and extending proximally (Hoeijmakers et al., 2012; Gondim et al., 2018). However, non-length-dependent and patchy symptoms (on the face, tongue, scalp, trunk) have also been reported (Hoeijmakers et al., 2012; Gondim et al., 2018). When present, negative symptoms include thermal sensory loss and numbness (Hoeijmakers et al., 2012).

Autonomic dysfunction alone or combined with sensory manifestations could also be the result of small fiber neuropathy (Bril and Katzberg, 2014). Although not a prominent feature of immune-mediated neuropathies related to SARDs, autonomic dysfunction can occur in SS, SLE and SSc (Adams et al., 2012). Autonomic dysfunction has clinical manifestations ranging from mild symptoms to pandysautonomia that can be life-threatening or severely affecting the quality of life. Clinical findings can be further classified into cardiovascular (at-rest tachycardia, orthostatic hypotension, silent myocardial ischemia), gastrointestinal (gastroparesis, diarrhea, constipation, fecal incontinence), genitourinary (neurogenic bladder, erectile dysfunction) and sudomotor manifestations (anhidrosis, excessive sweating, heat intolerance) as well as pupillary abnormalities (i.e. Argyll Robertson pupils) (Adams et al., 2012).

Cranial neuropathy might be encountered in SARDs in an isolated fashion or a multifocal distribution, but its etiopathogenesis is not always related to the vasculitic process–optic and olfactory nerves might be affected by granulomatous invasion, whereas basilar meningitis or other inflammatory processes might involve the rest of the cranial nerves (Wludarczyk and Szczeklik, 2016).

Acute motor axonal neuropathy (AMAN) and acute motor and sensory axonal neuropathy (AMSAN) are two variants of GBS rarely reported in SARDs, notably in LES (Santiago-Casas et al., 2013; Thakolwiboon et al., 2019). Acute motor axonal neuropathy presents as acute ascending symmetrical quadriparesis with occasional preservation of deep tendon reflexes, whereas AMSAN is a more severe form of AMAN, with superimposed sensory symptoms. It is important to recognize these entities since they might require a combination of IV immunoglobulin or plasma exchange and immunosuppressants (Ubogu et al., 2001; Santiago-Casas et al., 2013).

Asymptomatic neuropathy is a controversial entity detected only by nerve conduction studies and/or nerve biopsy performed in patients with systemic symptoms and laboratory findings suggestive of a systemic vasculitis. As already mentioned, neuropathy could serve as a diagnostic criterion for systemic vasculitides. Kurt et al. identified 21 patients with asymptomatic, biopsy-proven vasculitic neuropathy, out of which 20 had diffuse neuropathy involving both legs and one had mononeuritis multiplex (Kurt et al., 2015). Similar to other studies, they reported a prevalence of 7.8% of asymptomatic vasculitic neuropathies among patients with biopsy-proven vasculitis. Sural nerve conduction studies and biopsy were abnormal in all the cases, indicating a high sensitivity of these methods. Interestingly, the majority of these patients had biopsy-proven active vasculitis (Kurt et al., 2015). These data indicate the relevance of searching for neuropathy in the setting of a systemic vasculitis.

### Types of Immune Axonal Neuropathy in Systemic Autoimmune Rheumatic Diseases

Axonal sensorimotor polyneuropathy is the main presentation of neuropathy in collagen vascular diseases (Bril and Katzberg, 2014) (Table 1).

**TABLE 1:** Types and frequency of immune-mediated axonal neuropathy in SARDs. Legend: +++ - > mostly found, ++ - > frequently encountered, + - > moderately found,/ - > rarely encountered, sar - > sarcoidosis, cryo - > cryoglobulinemia, PNS - > peripheral nervous system.

Clinical presentation	SLE	RA	SS	aPL	SSc	Sar	BD	PAN	EGPA	GPA	MPA	Cryo
Sensorimotor polyneuropathy	+++	+++	⁄	+++	++	—	⁄	+++	+	+++	+	++
Pure sensory polyneuropathy	+	+++	⁄	++	++	—	⁄	—	+	—	—	+
Mononeuritis multiplex	++	++	⁄		⁄	—	⁄	+++	+++	+++	+++	+++
Sensory neuronopathy	⁄	—	+++	—	—	—	—	—	—	—	—	—
Small fiber neuropathy (sensory)	+	+	+++	++	+++	++	—	—	—	—	—	—
Autonomic involvement	+	+	+++	+++	+++	++	⁄	—	—	—	—	—
Cranial neuropathy	+		/(5th)		++(5th)	+++(7th)	⁄	+		+	+	
GBS axonal variants	⁄	—	⁄	—	—	—	—	—	—	—	—	—
PNS involvement	20–27%	15–70%	30–45%	35%	—	4–20%	—	65–85%	65–80%	5–50%	6–75%	30–70%

Systemic lupus erythematosus affects the central and peripheral nervous system either independently or simultaneously (Bril and Katzberg, 2014)–neuropsychiatric events attributed to SLE emerge as primary manifestations of SLE and should be distinguished from complications of the disease (e.g. hypertension) and its therapy (e.g. diabetes, infections) or concurrent neuropsychiatric disease not related to lupus (Hanly, 2014). All axonal neuropathy phenotypes have been reported in SLE patients, namely axonal sensorimotor polyneuropathy, mononeuritis multiplex, cranial neuropathy, small fiber neuropathy, sensory neuronopathy (ganglionopathy), AMAN and AMSAN, reflecting the diverse mechanisms of neuropathy in this disease (Bril and Katzberg, 2014; Hanly, 2014). However, axonal sensorimotor polyneuropathy is the main presentation, followed by mononeuritis multiplex (Mizisin and Weerasuriya, 2011). Sensory loss involving vibratory and position senses is usually more prominent than motor deficit in axonal sensorimotor polyneuropathy (Ropper et al., 2014). Although axonal degeneration is the cardinal histopathological pattern (70–80%), demyelination can also occur (20%) (Ropper et al., 2014; Blaes, 2015). Neuropathy is rarely the inaugural manifestation in SLE and usually occurs in the advanced stages of the disease (Ropper et al., 2014) and correlates with disease activity (Mizisin and Weerasuriya, 2011). Interestingly, AMAN and AMSAN have been reported as initial manifestations of SLE in 4 cases and preceding the diagnosis of SLE in 2 cases (Thakolwiboon et al., 2019).

Rheumatoid arthritis involves the peripheral nervous system in up to 75% of cases. Neuropathy usually takes the form of an aggressive axonal sensorimotor polyneuropathy, but mononeuritis multiplex also manifests in up to 8% of patients with RA (Bril and Katzberg, 2014). One study found pure sensory polyneuropathy to be the most prevalent pattern of neuropathy in RA patients (Agarwal et al., 2008) and another one reported pure motor polyneuropathy in 15 patients with RA (Kaeley et al., 2019). Mononeuritis multiplex is sometimes not easily differentiated from entrapment mononeuropathy (secondary to thickened tendons and destructive joint lesions) or drug-induced neuropathy (Blaes, 2015). Neuropathy mostly occurs in strongly seropositive patients (Ropper et al., 2014) in the advanced stages of the disease (Bril and Katzberg, 2014), accompanying other extraarticular manifestations such as rheumatoid nodules and purpura (Ropper et al., 2014).

Sjögren’s syndrome is an autoimmune disease mainly affecting the exocrine glands, therefore causing xerophthalmia and xerostomia. Extraglandular manifestations include peripheral neuropathies (2–64%), but only few of them are vasculitic (Blaes, 2015). SS has a wide spectrum of neuropathy phenotypes. Small fiber neuropathy is the most frequent finding, presenting with burning pain if sensory fibers in the skin are damaged and dysautonomia provided that autonomic fibers are involved (Bril and Katzberg, 2014). However, autonomic dysfunction is rarely inaugural and usually accompanies sensory symptoms (Adams et al., 2012). Sensory neuronopathy leading to sensory ataxia is also a common finding, as opposed to sensorimotor polyneuropathies that only occur in 1% of patients with SS (Bril and Katzberg, 2014); cranial neuropathy (especially trigeminal sensory neuropathy) and mononeuritis multiplex have been reported even more rarely (Bril and Katzberg, 2014).

Primary antiphospholipid syndrome associates neuropathy in up to 35% of cases according to Santos et al. (Santos et al., 2010). In their group of patients, the most common phenotype was sensorimotor polyneuropathy, followed by sensory polyneuropathy and carpal tunnel syndrome (Santos et al., 2010). Interestingly, most of the patients with electrophysiological findings compatible with neuropathy had no clinical symptoms or signs (Santos et al., 2010). Schofield identified 22 patients with aPS and autonomic neuropathy as the inaugural manifestation, out of which 71% also had sensory small fiber neuropathy (Schofield, 2017). Autonomic involvement was associated with significant thrombotic risk (Schofield, 2017).

Systemic sclerosis and localized scleroderma affect the peripheral nervous system in up to 30% of cases. Sensory symptoms and dysautonomia (including gut motility disturbance) are the most frequent complaints of patients with SSc (Blaes, 2015). Cranial nerve involvement occurs in almost 8% of patients with localized scleroderma, encompassing 7th, 3rd and 6th nerve palsies as well as trigeminal neuropathy (Amaral et al., 2013). Systemic sclerosis mostly features trigeminal neuropathy (16.52%), peripheral sensorimotor polyneuropathy (14.25%) and entrapment neuropathies (9.25%) and, to a lesser extent, symptomatic carpal tunnel syndrome (6.65%), ulnar nerve involvement (3.39%), mononeuritis multiplex (1.81%) and facial nerve damage (1.58%) (Amaral et al., 2013). Brachial plexopathy (0.68%), lumbar plexopathy (0.44%), 8th nerve (0.44%) as well as other cranial nerve (e.g. 6th, 9th, 12th nerve) involvement are rarely found in SSc (Amaral et al., 2013). Autonomic neuropathies have been reported in up to 80% of patients with SSc. Parasympathetic underactivity and sympathetic overdrive were most commonly encountered and could precede visceral fibrosis (Amaral et al., 2013). Nevertheless, Paik et al. (Paik et al., 2016) found that 35% of patients with SSc and peripheral neuropathy had an alternative cause for the nerve impairment, recommending screening for other etiologies since they might be reversed or potentially treatable.

In sarcoidosis, cranial neuropathy is the most common manifestation of peripheral nervous system involvement (Lacomis, 2011). Any cranial nerve could be affected, but facial nerves are most frequently involved, sometimes bilaterally (Lacomis, 2011). Heerfordt syndrome is a pathognomonic manifestation of sarcoidosis, including facial palsy combined with uveitis, chronic fever and parotid enlargement (Bril and Katzberg, 2014). Apart from the granulomatous infiltration and compression of nerves along their course, basilar leptomeningitis can also be responsible for cranial nerve palsies (Lacomis, 2011). Basilar leptomeningitis can have a monophasic, chronic or relapsing course, usually with a good outcome (Lacomis, 2011). Peripheral neuropathy occurs in 4–20% of patients with sarcoidosis and all axonal subtypes have been reported (Said et al., 2002; Lacomis, 2011). Out of these, mononeuropathies affecting ulnar and peroneal nerves were the most frequent (Lacomis, 2011). Peripheral neuropathy can occur at any stage of disease (Lacomis, 2011).

In BD, neurological involvement carries a bad prognosis (Emam, 2019). Cranial neuropathy and peripheral neuropathy are atypical presentations of BD and other plausible causes should be excluded in order to link them to BD, especially iatrogenic effects of colchicine or thalidomide (Siva and Saip, 2009; Dutra LAaB, 2016). Birol et al. found subclinical neuropathy in almost 77% of BD patients, with lower extremity nerves being more affected, especially sural and peroneal nerves (Birol et al., 2004). Sensory and motor components were equally involved according to the electrophysiological studies (Birol et al., 2004). Sensorimotor polyneuropathy, mononeuritis multiplex, autonomic dysfunction, sensory neuropathy with recurrent episodes of myositis and cranial neuropathy can sporadically occur in BD (Siva and Saip, 2009; Dutra LAaB, 2016).

As previously mentioned, primary systemic vasculitides are idiopathic inflammatory diseases of blood vessels that can virtually affect any organ or tissue in the body, including the peripheral nervous system (Table 1).

Polyarteritis nodosa is a primary systemic necrotizing vasculitis of the medium-sized vessels. Absence of glomerulonephritis, ANCA and small vessel involvement are its distinctive features. PAN might be either idiopathic or triggered by viral infections such as hepatitis B virus (HBV) (Collins et al., 2013). The peripheral nervous system is the most frequent target, the prevalence of neuropathy in HBV-associated PAN being even higher than in the idiopathic form (Collins et al., 2013). Neuropathy is the first clinical event in one third of PAN patients (Bril and Katzberg, 2014). Up to 72% of patients present with mononeuritis multiplex (mainly of the lower extremity) and less than 2% associate cranial nerve palsy (Hernández-Rodríguez et al., 2014). Mononeuritis multiplex in PAN usually has a sudden onset with pain or numbness in the distal part of a nerve and sensory or motor deficit in the distribution of the same nerve (Ropper et al., 2014). As mononeuritis multiplex in PAN features many small nerve infarctions, it might seem symmetrical and generalized, resembling a polyneuropathy (Ropper et al., 2014).

Eosinophilic granulomatosis with polyangiitis, formerly known as Churg Strauss syndrome, is an ANCA-associated primary systemic vasculitis (usually against myeloperoxidase: p-ANCA) of the small-to-medium sized vessels that presents with asthma, paranasal sinus abnormalities, non-fixed pulmonary infiltrates, blood eosinophilia and tissue eosinophilic invasion. Peripheral nervous system involvement occurs in up to 65% of patients and is preceded by constitutional symptoms, giving rise to different phenotypes, particularly mononeuritis multiplex, mononeuropathy or polyneuropathy (Bril and Katzberg, 2014; Ropper et al., 2014). The onset is often acute, with tingling or painful paresthesia in the distal parts of the lower limbs (Koike and Sobue, 2013).

Granulomatosis with polyangiitis, formerly known as Wegener’s granulomatosis, is also an ANCA-associated primary systemic vasculitis (usually against proteinase 3: c-ANCA) of the small-to-medium sized vessels that primarily affects the upper and lower respiratory tract as well as renal glomeruli. Vasculitic neuropathy is the first clinical presentation in 8% of GPA patients (Suppiah et al., 2011) and is distributed as mononeuritis multiplex in most of the cases (Blaes, 2015). A distinctive feature of peripheral nervous system involvement is lower cranial nerve mononeuropathy (Ropper et al., 2014).

Conversely, MPA is a pauci-immune necrotizing vasculitis of lung and kidney capillaries mainly associated with p-ANCA that does not elicit granulomatous inflammation. Motor polyneuropathy and sensorimotor polyneuropathy are as frequent as in GPA (7%), whereas pure sensory neuropathy and cranial nerve palsy are far less commonly encountered (Suppiah et al., 2011). Instead, the clinical course of MPA shares many similarities with EGPA, with sensory impairment distributed as mononeuritis multiplex in the initial phase (Koike and Sobue, 2013).

Cryoglobulinemia is a paraproteinemic state that leads to a small-vessel immune-complex-mediated vasculitis. Its main clinical features are purpura, arthralgia and renal involvement. It might be classified into type I cryoglobulinemia associated with monoclonal immunoglobulins, type II cryoglobulinemia with both monoclonal and polyclonal immunoglobulins and type III cryoglobulinemia with polyclonal IgG. Type III is most frequently accompanied by peripheral nervous system involvement and is usually the consequence of a systemic disease, either an infection or a noninfectious inflammatory disorder (Bril and Katzberg, 2014). Painful mononeuritis multiplex is the main clinical presentation, but aggressive sensorimotor polyneuropathy is also encountered (Bril and Katzberg, 2014; Blaes, 2015). The course of the asymmetrical form is slower than in other diseases, with long “silent” periods between the attacks of mononeuropathy (Ropper et al., 2014). Interestingly, there is no relationship between the severity of neuropathy and the titer of serum cryoglobulins (Ropper et al., 2014).

## Paraclinical Findings

Although a peripheral nerve disorder is suspected based on history and physical examination, paraclinical tests are essential in making the final diagnosis of immune-mediated axonal neuropathies. Table 2 provides information on laboratory tests and their utility in the diagnosis of immune-mediated neuropathies related to SARDs (Table 2).

**TABLE 2:** Paraclinical tests and their utility in patients with SARDs and immune-mediated axonal neuropathies related to SARDs. Some tests are mandatory (routine blood tests, differential diagnosis of axonal neuropathies, electrophysiological testing), whereas others should be performed in selected cases, according to the clinical suspicion. Legend: abs - > antibodies, HbA1c - > glycated hemoglobin.

Paraclinical tests		Utility
Blood and urine tests	Complete blood count; erythrocyte sedimentation rate; C-reactive protein; fibrinogen; creatinine; blood urea nitrogen; electrolytes; urinalysis; transaminases; gamma-glutamyl transpeptidase; alkaline phosphatase; lactate dehydrogenase; creatine kinase	Activity and expansion of SARDs
	c-ANCA and *p*-ANCA; antinuclear abs; anti-double-stranded DNA abs; C_3_; C_4_; cryoglobulins; rheumatoid factor; anti-CCP abs; ACE; anti-ro/SSA and anti-La/SSB abs	Diagnosis of SARDs
	Hepatitis B surface antigen; hepatitis C abs; anti-HIV abs	Differential diagnosis of SARDs; Etiological diagnosis of neuropathy
	Fasting glucose; HbA1c; vitamin B_12_; thyroid hormones; immunofixation electrophoresis; immunogram; β_2_ microglobulin; anti-tissue transglutaminase abs; anti-borrelia burgdorferi abs; VDRL; toxicology testing; spot urine for porphobilinogen and total porphyrins	Differential diagnosis of immune-mediated axonal neuropathy related to SARDs
Electrophysiological testing	NCS; EMG	Diagnosis of axonal neuropathy
		Fiber type involvement
		Distribution of neuropathy
		Severity of neuropathy
	Nociceptive-evoked potentials; microneurography	Function of somatic small fibers
Imaging methods	Optical coherence tomography; sinus x-ray; echocardiography; abdominal ultrasound/CT; joint ultrasonography; contrast-enhanced brain MRI	Expansion of SARDs; Diagnosis of SARDs
	High resolution nerve sonography	Complementary role in mononeuropathy diagnosis
	Contrast-enhanced spine and limb MRI	Enhancement of nerve roots, plexuses and nerves can occur in sarcoidosis; hyperintense T2-weighted lesions and volumetric reduction of posterior columns can occur in chronic sensory neuronopathy
	Corneal confocal microscopy	Possible use in the diagnosis of sensory small fiber neuropathy
CSF analysis	NFL (also from serum)	Might differentiate active vasculitic neuropathy from non-vasculitic neuropathy
	Glucose (CSF:serum ratio); protein; cell count	Pleocytosis and hypoglycorrhachia could be found in sarcoidosis
		Pleocytosis might occur in AMAN
	Angiotensin-converting enzyme	Elevated values might occur in sarcoidosis
Biopsy	Nerve biopsy or combined nerve-muscle biopsy	“Gold standard” for the diagnosis of vasculitic neuropathy; should be performed when there is a high suspicion of vasculitis
	Skin biopsy with quantification of IENFD	Diagnosis of small fiber neuropathy
		Small fiber neuropathy progression and response to treatment
		Etiology of small fiber neuropathy
	Dorsal root ganglia excisional biopsy	Rarely performed for the diagnosis of sensory neuronopathy
Autonomic testing	Ewing battery test; QSART; TST; SSR; SVR; ARFS	Cardiovascular autonomic reflex and sudomotor and vasodilation function of autonomic fibers

### Blood Tests and Imaging Methods

Blood and urine tests should be performed in suspected SARDs, both for establishing the diagnosis (and excluding differential diagnoses) and assessing disease activity and expansion. Routine blood tests and urinalysis could identify relevant findings such as cytopenia, eosinophilia, inflammatory biomarkers, proteinuria, elevated transaminases, cholestasis and elevated creatine kinase levels, whereas immunological tests such as autoantibodies support the diagnosis of SARDs (Bril and Katzberg, 2014; Blaes, 2015).

Relevant findings for SARDs diagnosis and expansion can also be detected by imaging modalities such as: optical coherence tomography–uveitis, optic neuropathy; sinus x-ray–sinusitis; chest x-ray/CT–chest infiltrates, lymphadenopathy, pleural effusion; echocardiography–pericardial effusion, Libman-Sacks endocarditis; abdominal ultrasound/CT–lymphadenopathy, hepatosplenomegaly; joint ultrasonography–arthritis, synovitis. A contrast-enhanced brain MRI might identify basal meningeal enhancement in sarcoidosis presenting with multiple cranial neuropathies (Pirau and Lui, 2020).

Nevertheless, we emphasize the fact that SARDs are usually diagnosed based on classification criteria (in the absence of diagnostic criteria), which means that exclusion of alternative diagnoses such as infections and malignancies is mandatory (Aggarwal et al., 2015). Moreover, other plausible causes for axonal neuropathy should be excluded before linking it to SARDs.

### Electrophysiological Studies

In immune axonal neuropathies, electrodiagnosis reveals an axonal process, identifies the type of the affected fiber and indicates the distribution, course and severity of the disease (Karvelas et al., 2013).

Axonal degeneration impairs its function, leading to failure in properly conducting electrical signals to the recording electrode. Reduced sensory nerve action potentials (SNAPs) and compound muscle action potentials (CMAPs) amplitudes subsequently emerge, usually in a sequential manner–the sensory nerves are the first ones to display electrophysiological abnormalities, possibly because of their lack of compensatory reinnervation, whereas CMAP amplitudes can be normal until 75% of axons are affected as there is collateral sprouting in the motor nerves. However, axonal neuropathies may fail to show abnormalities on nerve conduction studies (NCS) in the early phases, exhibiting spontaneous muscle activity and aberrant motor unit action potential morphology on needle electromyography (EMG) instead. Since demyelinating features such as increased latencies, prolonged F waves or diminished conduction velocities might occur as a consequence of fast conducting fibers loss or demyelination secondary to prominent axonal loss, electrophysiological distinction between axonal and demyelinating processes might be a challenge (Karvelas et al., 2013). In this regard, Tankisi et al. (Tankisi et al., 2005) proposed a series of criteria for electrophysiological classification of polyneuropathies. According to them, a primarily axonal process requires the presence of at least two nerves (sensory and/or motor) fulfilling the criteria for axonal loss, namely a decrease in SNAP or CMAP amplitude with at least 2.5 standard deviations (SD) and a slight reduction in conduction velocity/distal motor latency by up to 2.5 SD and consistent EMG findings (Tankisi et al., 2005).

Standard electrophysiological studies indicate the fiber type involvement - motor or large sensory - by analyzing the SNAPs and CMAPs latencies and amplitudes. They do not directly assess the small sensory fibers responsible for pain/burning sensation in the extremities or small autonomic fibers and are expected to be normal in isolated involvement of these nerve fibers (Karvelas et al., 2013). An asymmetrical axonal sensory neuropathy without distal worsening gradient toward the legs is suggestive of sensory neuronopathy (ganglionopathy) (Martinez et al., 2012). Up to 18% of the patients also have reduced CMAP amplitude, especially at peroneal and tibial nerves, whereas EMG is usually normal in sensory neuronopathy (Martinez et al., 2012).

The distribution of neuropathy gives important clues regarding its etiopathogenesis. In mononeuritis multiplex multiple single nerves are affected either simultaneously or serially in an asymmetrical pattern. Chronic and severe vasculitis leads to axonal loss in multiple areas of a single nerve with eventual involvement of multiple nerves mimicking a distal symmetric polyneuropathy. In the initial phases of a distal symmetric neuropathy, the amplitudes of SNAPs and CMAPs are diminished in the distal lower limbs. As the disease progresses, similar abnormalities are likely to be found in the distal upper limbs. Moreover, a neurogenic pattern can be spotted on EMG.

The course and severity of neuropathy are assessed indirectly by electrophysiological studies. The severity of axon loss is well correlated with electrophysiological findings, but not with symptoms. Motor deficit is not clinically evident until 50% of the axons in the motor unit are damaged, but denervation with spontaneous muscle activity can be detected on EMG after minimal axonal degeneration. On the other hand, chronic neuropathies are likely to have collateral sprouting with fiber reinnervation expressed as abnormal motor unit action potential morphology (increased amplitude, duration and polyphasia) on EMG.

Since NCS are normal in small fiber neuropathy, other tests have been employed to assess the function of somatic and/or autonomic small fibers. Quantitative sensory testing (QST) assesses temperature thresholds by two methods: the method of levels and the method of limits (Hoeijmakers et al., 2012). In small fiber neuropathy, QST high thresholds are expected. However, QST specificity is low since central nervous system disorders such as multiple sclerosis and stroke might impair the results (Hoeijmakers et al., 2012). Another limit of QST is that the patient needs to be alert and cooperative (Hoeijmakers et al., 2012). Nociceptive-evoked potentials (laser-evoked potentials, contact-heat evoked potentials, pain-related evoked potentials, intraepidermal electrical stimulation) are elicited by selective stimulation of Aδ fibers and/or C-fibers (Hoeijmakers et al., 2012). Poor responses are correlated with the severity of the disease (Hoeijmakers et al., 2012). Microneurography provides direct measurement of sympathetic activity, but requires expert investigators and cooperative patients (Hoeijmakers et al., 2012). Autonomic testing such as the Ewing battery test for cardiovascular autonomic reflex and Quantitative Sudomotor Axon Reflex Test (QSART), Thermoregulatory Sweat Test (TST), Sympathetic Skin Response (SSR), Skin Vasomotor Reflex (SVR) as well as Axon Reflex Flare Size (ARFS) for sudomotor and vasodilation function could be used for assessing autonomic small fiber function (Hoeijmakers et al., 2012).

### Cerebrospinal Fluid Analysis

Cerebrospinal fluid (CSF) analysis is not commonly recommended in neuropathies since it has a low diagnostic yield (except for demyelinating neuropathies) (England et al., 2009). However, serum neurofilament light (NFL), a structural protein specific to neurons that was previously proven indicative of axonal damage in central nervous system disorders such as multiple sclerosis, has displayed a 100% specificity and 82% sensitivity for a cut-off value of 155 pg/ml in discriminating between active vasculitic neuropathy and non-vasculitic neuropathy or systemic vasculitis without neuropathy (Bischof et al., 2018). Mariotto et al. (Mariotto et al., 2018) showed that serum and CSF NFL levels are increased not only in vasculitic neuropathies, but in many other types of acquired neuropathies irrespective of neurophysiological findings or clinical subtypes. Furthermore, they proved that serum NFL is correlated with disease activity and disability progression, subsequently extending the previous findings and suggesting a potential role for this biomarker in monitoring axonal damage and treatment efficiency in peripheral nervous system disorders (Mariotto et al., 2018). In neurosarcoidosis, CSF analysis occasionally reveals elevated angiotensin-converting enzyme, pleocytosis and hypoglycorrhachia (Bril and Katzberg, 2014). In AMAN and AMSAN, CSF pleocytosis can occur (Bril and Katzberg, 2014).

### Nerve Sonography and Magnetic Resonance Imaging

High resolution nerve sonography directly visualizes the peripheral nerves and provides data regarding their morphology. In addition to the well-known advantages of echography such as non-invasivity, low price and good tolerability, it provides an easy access to small fibers and many peripheral nerves since they display a superficial course, as well as a rapid assessment of a long nerve course (Goedee et al., 2013). Its complementary role in diagnostic approach has already been validated in mononeuropathies, but available data regarding polyneuropathies are still lacking. Information on vasculitic neuropathy is even more scarce, sometimes being obtained indirectly–for instance, Rajabally et al. (Rajabally et al., 2012) found that distal median nerve is significantly thicker in chronic inflammatory demyelinating polyneuropathy compared to vasculitic neuropathy. Nodora et al. (Nodera et al., 2006) reported a patient with vasculitic neuropathy who had multiple nerve hypertrophy, as suggested by bilateral enlargement (i.e. increased cross-sectional area) of the median, ulnar, tibial and cervical nerve roots that diminished after steroid treatment. Ito et al. (Ito et al., 2007) proved tibial nerve thickening and hypoechogenicity (possibly secondary to intraneural edema) in eight patients with vasculitic neuropathy. Data related to the size of fascicles, thickness of the epineurium or nerve vascularization are unavailable in the aforementioned studies. However, high resolution nerve sonography is to be considered in axonal neuropathies and should benefit from further quality research in order to be validated as a diagnostic tool, especially since electrophysiological studies are often not well tolerated because of intense pain (Goedee et al., 2013).

In sarcoidosis, apart from the basal meningeal enhancement already mentioned, contrast enhancement of nerve roots, plexuses and nerves can sometimes be identified on spine and limb MRI (Pirau and Lui, 2020). In chronic sensory neuronopathy, hyperintense T2-weighted lesions and volumetric reduction of posterior columns are evident due to dorsal root ganglia neuronal destruction with subsequent degeneration of their central projections (Martinez et al., 2012).

### Nerve Biopsy

**FIGURE 2:**
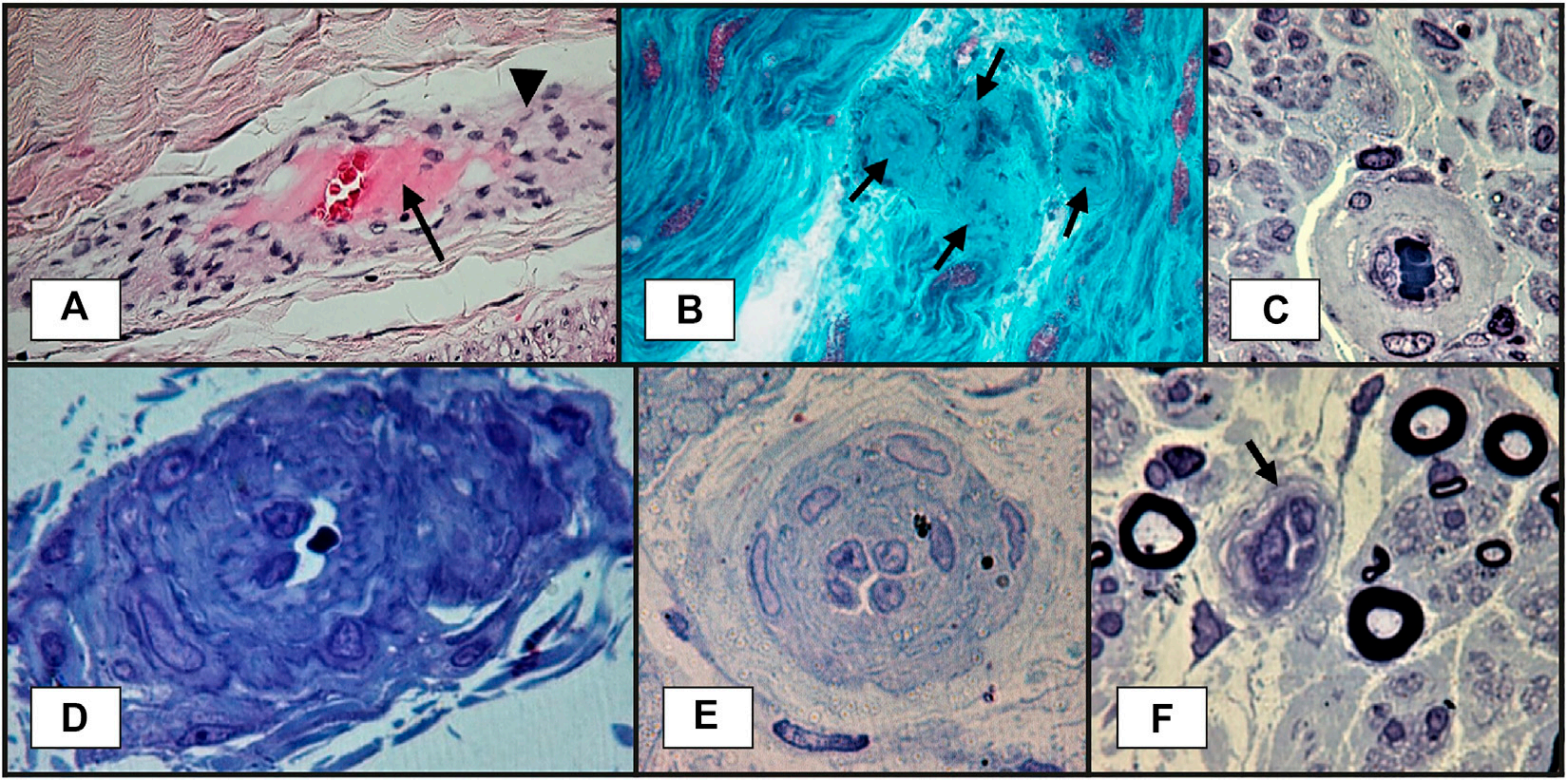
Sural nerve biopsy showing vasa nervorum lesions suggestive of vasculitic neuropathy: **(A)**–transmural inflammation (arrowhead) and fibrinoid necrosis: thick layer in the center of the vessel, near lumen, in pink (arrow). This section is slightly oblique, so the vessel wall is elongated (paraffin embedded tissue section, H&E staining); **(B)**–blood vessel wall thickening, with marked luminal narrowing and presumptive fibrinoid necrosis (arrows) (longitudinal cryosection, modified Gӧmӧri trichrome staining); **(C)**–thickened vessel wall in endoneurium (semithin section, epon embedding tissue, toluidine blue staining); **(D)**–a vessel with abnormal structure and thickened wall in epineurium. A narrowed lumen is observed (semithin section, epon embedding tissue, toluidine blue staining); **(E)**–a vessel with reactive endothelial cells, multilayered basal membrane and very narrow lumen (semithin section, epon embedding tissue, toluidine blue staining); **(F)**–a vessel with reactive endothelial cells and narrow lumen (arrow) (semithin section, epon embedding tissue, toluidine blue staining).

**FIGURE 3:**
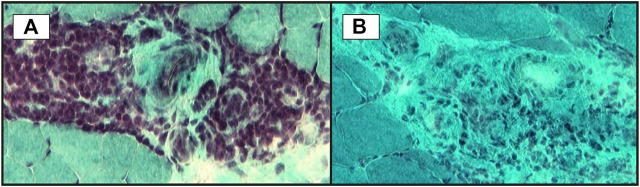
Small vessels inflammation in skeletal muscle biopsy: **(A)**–significant perivascular inflammatory infiltrate; **(B)**–microvasculitis: some small vessels with perivascular inflammatory infiltrate (transversal cryosections, modified Gӧmӧri trichrome staining).

**FIGURE 4:**
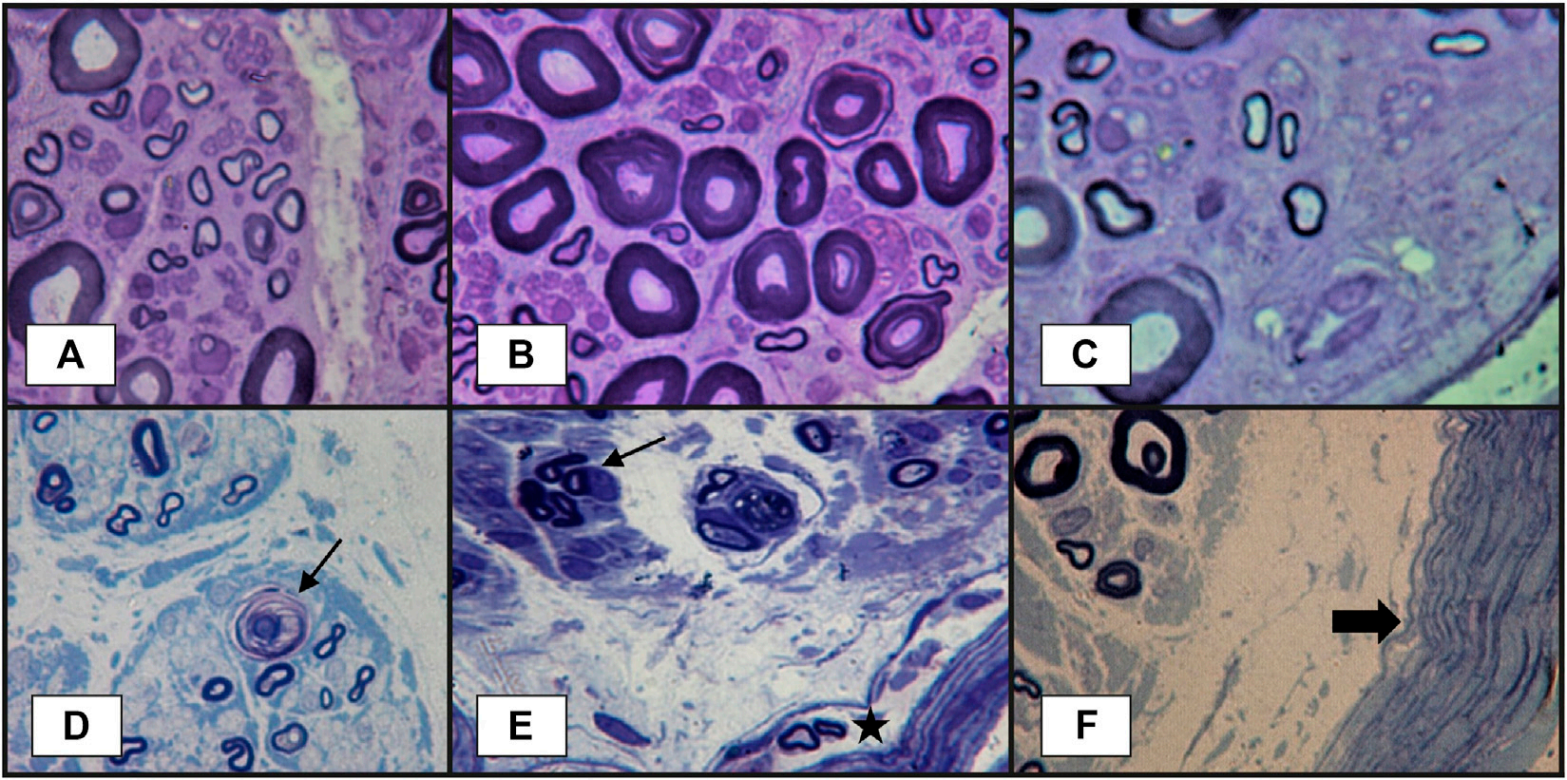
Peripheral nerve lesions in vasculitic neuropathy: **(A–C)**–transverse semithin sections of sural nerve biopsy in a patient with severe acute axonal neuropathy, showing findings that are highly suggestive of vasculitic neuropathy: focal fiber damage in the same nerve fascicle with axonal degeneration in different zones (three images from the same semithin section), loss of large-caliber axons; **(D)**–another case more severely affected, with focal axonal loss, axonal degeneration (arrow) and subperineurial edema. Other abnormalities observed in vasculitic peripheral neuropathy, but without specificity, could be **(E)**–axonal los, axonal regeneration represented by the presence of a small group of axons with same caliber (arrow), some abnormal small myelinated axons in perineurium (star); **(F)**–thickened perineurium (thick arrow) with subperineurial marked edema (semithin sections, epon embedding tissue, toluidine blue staining).

**FIGURE 5:**
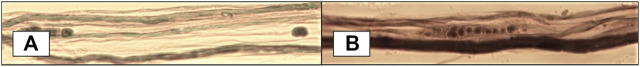
Teased fiber studies showing axonal degeneration in dissociated myelinated fibers - fibers with myelin spheres and ovoids along with normally myelinated axons (osmium tetroxide).

Nerve biopsy is indicated whenever there is a high suspicion of vasculitis but no supportive evidence from other paraclinical tests (Bril and Katzberg, 2014; Blaes, 2015). The classical histopathological findings of vasculitic neuropathy are: inflammatory infiltration of vasa nervorum with wall destruction, fibrinoid necrosis of the tunica media with fragmentation of the internal elastic lamina and centrofascicular axonal degeneration (Vallat et al., 2009; Collins et al., 2013). In older lesions, the fibrinoid necrosis is replaced by extensive fibrosis, but inflammatory cells are still present at the periphery (Vallat et al., 2009). There are three histopathological degrees of certainty for the diagnosis of vasculitic neuropathy: definite, probable and possible. The diagnosis of vasculitic neuropathy is certain when intramural vasa nervorum inflammation is associated with vascular wall damage in the absence of vasculitic mimicker (Collins et al., 2010). Vascular wall damage is reflected by either active lesions such as “fibrinoid necrosis, loss/disruption of endothelium, loss/fragmentation of internal elastic lamina, loss/fragmentation/separation of smooth muscle cells in media, acute thrombosis, vascular/perivascular hemorrhage or leukocytoclasia” or chronic lesions with signs of healing/repair, namely “intimal hyperplasia, fibrosis of media, adventitial/periadventitial fibrosis or chronic thrombosis with recanalization” (Collins et al., 2010). Some supportive criteria for the diagnosis of vasculitic neuropathy (also considered pathologic predictors of definite vasculitic neuropathy) have also been proposed, specifically “neovascularization, endoneurial hemorrhage, focal perineurial inflammation/degeneration/thickening, injury neuroma, microfasciculation, swollen axons filled with organelles and other axonal changes of acute ischemia” (Collins et al., 2010). Probable vasculitic neuropathy is suspected whenever predominant axonal changes occur in addition to both perivascular/vascular inflammation and vascular damage or “vascular deposition of complement/IgM/fibrinogen/hemosiderin, asymmetrical/multifocal nerve fiber loss/degeneration, prominent active axonal degeneration, myofiber necrosis, regeneration/infarcts in concomitant peroneus brevis muscle biopsy” (Collins et al., 2010). Predominant axonal changes associating either vascular inflammation, vascular damage or pathologic predictors of definite vasculitic neuropathy stand for a possible vasculitic neuropathy (Collins et al., 2010). Figures 2–5 reveal some of the features commonly encountered in vasculitic neuropathy.

Histological proof is the “gold standard” for the diagnosis of vasculitic neuropathy. However, superficial nerve biopsy (sural nerve) has a sensitivity of only 50%, whereas combined nerve-muscle biopsy (superficial peroneal nerve/peroneus brevis muscle or sural nerve/anterior tibialis or gastrocnemius muscle) adds a 5.1% additional yield in clinically suspected vasculitic neuropathy and 15% in established vasculitic neuropathy (Collins et al., 2010; Vrancken et al., 2011). In this setting, histopathological predictors of vasculitis have been looked for. Immune deposits (immunoglobulin, complement, fibrinogen) have been detected by electron microscopy in epineurial vessel walls in up to 80% of patients with vasculitic neuropathy, but their specificity has been subject of debate since their presence might be the consequence of the disruption of blood-nerve interface. Collins et al. (Collins et al., 2013) hypothesized that a larger amount of immune proteins in the vessel walls might be more specific for a vasculitic process, hence a specific diagnosis might require a laboratory method that fails to identify low concentrations of these proteins. Therefore, they evaluated a cohort of patients with suspected vasculitic neuropathy undergoing combined nerve-muscle biopsy with direct immunofluorescence analysis (a less sensitive method) for detection of IgG, IgM and C_3_ and demonstrated a 92% specificity of this method for the diagnosis of vasculitic neuropathy (Collins et al., 2010).

Skin biopsy is increasingly being used as an additional test for the diagnosis of small fiber neuropathy (Hoeijmakers et al., 2012). It employs morphometric evaluation of intraepidermal nerve fibers (the distal ends of axons originating from dorsal root ganglia and trigeminal ganglion that cross the dermo-epidermal junction and reach the epidermis) density (IENFD). Intraepidermal nerve fibers density is significantly reduced in small fiber neuropathy (Tesfaye et al., 2010). In addition to decreased IENFD, degenerative changes of IENF and dermal fibers could also be spotted (Hoeijmakers et al., 2012). Moreover, skin biopsy can also be used to evaluate autonomic structures and innervation in sweat glands and arrector pili muscles (Hoeijmakers et al., 2012). In diabetes mellitus and toxic neuropathies, IENF loss is an early feature of the disease, progresses with disease severity and may repair with proper management (Tesfaye et al., 2010; Hoeijmakers et al., 2012). Since damage of small nerve fibers is not disease-specific, these findings could also apply to immune-mediated small fiber neuropathy related to SARDs (Hoeijmakers et al., 2012). Skin biopsy can also identify the underlying cause of small fiber neuropathy when concurrent conditions such as diabetes mellitus emerge (Hoeijmakers et al., 2012). Perivascular inflammation and vascular injury point to SLE rather than diabetes mellitus (Hoeijmakers et al., 2012). Nevertheless, equivocal clinical-histological correlations have been reported, emphasizing the need for an accurate history taking and thorough neurological examination. For instance, decreased IENFD might arise as a result of distal damage to large sensory fibers (Gondim et al., 2018), but symptoms of small fiber involvement and normal NCS are highly suggestive of small fiber neuropathy (Levine, 2018). Corneal confocal microscopy is a non-invasive method that might be useful in the diagnosis of small fiber neuropathy (Hoeijmakers et al., 2012). Corneal fiber density seems to be inversely correlated with IENF loss in distal leg and disease severity (Hoeijmakers et al., 2012).

Dorsal root ganglia excisional biopsy with histological analysis reveals neuronal loss, the Nageotte nodules and mononuclear infiltrates in sensory neuronopathy (Martinez et al., 2012). However, this biopsy is rarely performed because is very invasive and requires trained neurosurgeons (Martinez et al., 2012).

We conclude that nerve biopsy is crucial for definite diagnosis in vasculitic neuropathies unless other examinations are conclusive. However, its low diagnostic yield in a phenotype not highly suggestive of a vasculitic neuropathy and the risks it implies (permanent hypoesthesia, wound infection and delayed healing or post-biopsy pain) require further research for identifying diagnostic biomarkers of vasculitic neuropathy with higher efficacy and safety profiles. Skin biopsy is the “gold standard” test for diagnosis of small fiber neuropathy. Dorsal root ganglia excisional biopsy is seldom performed for diagnosing sensory neuronopathy. Nerve biopsy is currently not recommended in other types of immune-mediated axonal neuropathy.

## Treatment

### Immunosuppressive and Immunomodulatory Treatment

Since neuropathy associated with SARDs is supposedly immune-mediated, its treatment aims for immunosuppression, likewise in other affected organs/tissues targeted by SARDs (lupus nephritis, rheumatoid lung disease, scleroderma-related interstitial lung disease, etc.). However, considering the low prevalence of systemic autoimmune diseases and the difficulty to conduct clinical trials, there is a scarcity of good evidence regarding their treatment, particularly when it comes to peripheral neurological involvement. Moreover, considering that peripheral neuropathy is usually overshadowed by life-threatening manifestations (pulmonary, renal, cardiac, central nervous system, severe thrombocytopenia or hemolytic anemia) or more disturbing symptoms (fever, polyarthritis, rash, malaise), treatment is generally directed toward these, whereas neuropathy is the main target only when it is an isolated manifestation or displays a severe and progressive course.

The guideline for the management of SLE with neuropsychiatric manifestations (Bertsias et al., 2010) states that “glucocorticoids alone or with immunosuppressive therapy have been used with good results (60–75% response rate). Intravenous immunoglobulin, plasma exchange and rituximab have been used in severe cases”, without listing any reference. Similarly, the recommendation regarding the treatment of peripheral neuropathy “Peripheral neuropathy: Combination therapy with glucocorticoids and immunosuppressive agents may be considered in severe cases” is rated with level A of evidence and class I recommendation (very strong), although the statement “may be considered” does not actually suggest a strong evidence. These features are inappropriate for a guideline and emphasize the lack of good quality data concerning immune-mediated neuropathy treatment. Furthermore, PubMed labels only one randomized controlled trial comparing IV cyclophosphamide with IV methylprednisolone in severe neurological manifestations of SLE, which reported only 7 patients with peripheral neuropathy out of 32 patients evaluated (Barile-Fabris et al., 2005). Additionally, there are only two retrospective observational studies regarding neurolupus, each evaluating 10 patients with neuropathy (Neuwelt et al., 1995; Fanouriakis et al., 2016), a restrospective case series of 10 patients with mononeuritis multiplex (Rivière et al., 2017) and various case reports. Generally, the cases describe patients who responded to an immunosuppressive drug or a combination therapy, sometimes following a lack of response to another immunosuppressive drug or combination, therefore providing no clear suggestion for a treatment algorithm, apart from the low evidence level (Constantin et al., 2020). Evidence is even more scarce for other SARDs that associate neuropathy, particularly RA, SS (Huma et al., 2020), PAN, ANCA-associated vasculitides or cryoglobulinemia.

Immunosuppressive treatment for immune-mediated axonal neuropathies related to SARDs consists of corticosteroids and cyclophosphamide, either in oral regimens or IV pulses. As in other organ involvement, rituximab and mycophenolate mofetil have been recently tried. Plasmapheresis is reserved for severe cases. Induction therapy for severe axonal immune-mediated neuropathy related to SARDs consists of prednisone 1 mg/kg (or methylprednisolone 7–15 mg/kg up to 1 g/day IV in severe cases of acute mononeuritis) plus either rituximab (off label) (375 mg/m^2^/week, four weeks) or cyclophosphamide (15 mg/kg IV every 2 weeks for three doses, then every 3 weeks, 6 or 7 pulses). Provided that cyclophosphamide or rituximab are not tolerated or are unavailable, azathioprine or mycophenolate could be used instead (the latter off label). The dose of azathioprine is 2–2.5 mg/kg if thiopurine methyltransferase (an enzyme required for azathioprine metabolism) is present. Otherwise, the drug is very toxic and is restricted; if the enzyme assays are unavailable or too expensive, azathioprine 50 mg/day is administered and blood cell count is checked after one week–absence of leukopenia means that the enzyme is present, requiring full dosage afterward. For mycophenolate mofetil the induction dose is 2–3 g/day for 6 months (dose for induction in lupus nephritis) (Hahn et al., 2012). The maintenance therapy includes either azathioprine (dose mentioned above) or mycophenolate mofetil (1–1.5 g b. i.d) (Ong et al., 2005; Hahn et al., 2012).

### Specific Advice for Each Disease/Manifestation

Systemic lupus erythematosus: cyclophosphamide pulse should be administered monthly instead of every 2 and then 3 weeks. If secondary aPL is confirmed, anticoagulants or antiplatelet agents should be used.

Rheumatoid arthritis: if the patient is already treated with DMARDs (either methotrexate or leflunomide) or biological therapy, ceasing them is recommended.

Sjögren’s syndrome: patients who do not respond adequately to symptomatic therapy could receive immunosuppressive medication, namely corticosteroids with azathioprine or rituximab. If cryoglobulinemic vasculitis (cryoglobulins can appear in SS) is suspected, immunosuppression should be more aggressive, with cyclophosphamide or rituximab. The same regimen applies for mononeuritis multiplex. In sensory neuronopathy, plasma exchange (5-9 sessions), IV immunoglobulin (3 cycles at 3 weeks interval) +/− rituximab, azathioprine (2–3 mg/kg/day) or TNFα antagonist infliximab (3 mg/kg in refractory cases) might have beneficial results (Martinez et al., 2012).

Sarcoidois: oral prednisone should be used in facial neuropathy (2 weeks) and peripheral neuropathy (4 weeks). For severe forms of neuropathy, IV methylprednisolone could be given for 3 days, followed by oral prednisone for 2–4 weeks, with subsequent tapering.

ANCA-associated vasculitides: acute-onset mononeuritis multiplex requires remission induction therapy as in vital organ involvement - high dose corticosteroids in combination with IV cyclophosphamide (3 pulses every 2 weeks, then 6–7 pulses every 3 weeks) or rituximab, as previously stated (Yates et al., 2016). In severe disease course, plasmapheresis could be added.

Polyarteritis nodosa: The “five factor score” that guides therapy in vasculitis does not include peripheral neuropathy. Corticosteroids and cyclophosphamide should be used for induction, while azathioprine or methotrexate maintain remission (De Virgilio et al., 2016). PAN secondary to hepatitis B virus infection requires treatment with lamivudine, short term use of corticosteroids and plasmapheresis in more severe cases.

Cryoglobulinemic vasculitis: rapidly progressive neuropathy is considered a feature of severe disease, requiring etiological treatment (most frequently, anti-hepatitis C) in addition to immunosuppressive therapy with rituximab alone or in combination with pulsed high-dose corticosteroids (instead of chronic use of low-medium doses); plasmapheresis should be performed in nonresponders, emergency situations or hyperviscosity syndrome (Pietrogrande et al., 2011).

Small fiber neuropathy: immunomodulatory therapy seems to show benefits in relieving symptoms (Hoeijmakers et al., 2012).

AMAN/AMSAN: apart from IV immunoglobulin or plasma exchange, immunosuppressants should also be used (IV methylprednisolone and high-dose cyclophosphamide).

Nevertheless, as previously mentioned, these recommendations are based on poor evidence (Benstead et al., 2014).

### Analgesic Treatment

There are no studies specifically addressing pain treatment in immune-mediated neuropathy. Therefore, recommendations are extrapolated from diabetic peripheral neuropathy trials: pregabalin has consistent evidence with class I recommendation, whereas other drugs like gabapentin, duloxetine, venlafaxine, amitriptyline, valproate, opioids and capsaicin are probably effective and should be considered for treatment (class II recommendation for pain in diabetic neuropathy) (Bril et al., 2011).

## Prognosis

Although immune axonal neuropathy does not affect patient survival in SARDs, it has a major negative impact on daily activities and functionality level, with subsequent decrease in life quality (Koike and Sobue, 2013; Naddaf and Dyck, 2015). Apart from the unpleasant subjective experience (pain, paresthesia) that interferes with daily activities and significantly alters the quality of life (mood, sleep, etc.), these patients can have balance impairment leading to falls due to sensory and motor deficits. As expected, patients with active vasculitic neuropathy are more likely to have constitutional symptoms, active disease and multivisceral involvement (Suppiah et al., 2011).

## Conclusion

### Final Remarks

About 30–50% of patients with systemic vasculitis and 5–93% of those with connective tissue disorders have peripheral nervous system involvement (Blaes, 2015; Snoussi et al., 2019), either as inaugural presentation or throughout the course of the disease. It is mandatory to recognize and properly assess immune axonal neuropathy since it might be the only manifestation of an aggressive disease or cause significant distress to the patient. Moreover, appropriately classifying the underlying disorder assists the clinician in choosing the right treatment and influence the prognosis. Discovering new diagnostic biomarkers with high safety and efficacy profiles would be valuable since the diagnosis is sometimes difficult and delayed.

### Future Perspectives

Considering the few trials directly addressing the effect of immunosuppression and immunomodulation on immune axonal neuropathy related to SARDs, current treatment strategies depend mainly on extrapolation from studies pertaining to other organ involvement such as lupus nephritis (Suppiah et al., 2011). High quality clinical research is required in order to provide indications and follow up guidelines for immunosuppressive therapies in immune axonal neuropathies associated with SARDs.
